# Assessment of Preprint Policies of Top-Ranked Clinical Journals

**DOI:** 10.1001/jamanetworkopen.2020.11127

**Published:** 2020-07-22

**Authors:** Dorothy S. Massey, Michelle A. Opare, Joshua D. Wallach, Joseph S. Ross, Harlan M. Krumholz

**Affiliations:** 1 Center for Outcomes Research and Evaluation, Yale New Haven Hospital, New Haven, Connecticut; 2 Department of Environmental Health Sciences, Yale School of Public Health, New Haven, Connecticut; 3 Section of General Medicine and the National Clinician Scholars Program, Department of Internal Medicine, Yale School of Medicine, New Haven, Connecticut; 4 Department of Health Policy and Management, Yale School of Public Health, New Haven, Connecticut; 5 Section of Cardiovascular Medicine, Department of Internal Medicine, Yale School of Medicine, New Haven, Connecticut

## Abstract

This cross-sectional study examines the preprint publication policies of 100 clinical journals with the highest impact factor.

## Introduction

The clinical research community has adopted the use of preprint servers, which provide outlets for preliminary reports of research that has not been peer-reviewed.^1^ Preprint servers support open scholarship, allow research to be disseminated quickly, offer opportunities for peer feedback before formal submission to a journal, and have been increasingly adopted by the biological, physical, and economic scientific communities.^2,3,4^ However, for preprint potential to be realized in clinical research, peer-reviewed journals must be willing to consider manuscripts that were previously posted on preprint servers (preprints) for publication. Because systematic information about contemporary clinical journal policies on preprints is lacking,^5^ our objective was to assess the preprint publication policies of the 100 clinical journals with the highest impact factors.

## Methods

This cross-sectional study followed the Strengthening the Reporting of Observational Studies in Epidemiology (STROBE) reporting guideline. For this study, we used InCites Journal Citation Reports (JCR) to identify journals across all fields with a 2018 journal impact factor greater than 5. We manually screened all identified journals by title and categories on JCR to find the 100 top-ranked clinical journals and included only those that publish original research.

For each qualifying journal, we checked a succession of resources to ascertain its editorial policy on preprints: the journal website; the publisher website; the Transpose Database^6^; and the first 10 pages of a Google search containing the journal name and the term *preprint*. Once a preprint policy was located, we classified each journal’s policy by the following categories: preprints allowed (if preprints will be considered for publication), case-by-case determination (if preprints are evaluated on an individual basis), and preprints prohibited (if preprints will not be considered for publication). Data were collected on April 23, 2020. We conducted descriptive analyses using Microsoft Excel (Microsoft Corporation).

## Results

Among the 100 top-ranked clinical journals, the median (interquartile range) impact factor was 13.7 (10.7-19.0). Most journals (86 [86%]) allowed preprints (Table 1). In contrast, 13 journals (13%) evaluated each preprint independently to determine whether to reject it on the basis of its prior preprint status (case-by-case determination). Only 1 journal (1%) had a policy that prohibited preprints (preprints prohibited). There was no association between the median impact factor and the category of preprint policy (Table 2).

**Table 1.** Preprint Policies by Category

Category	No. of journals (N = 100)	Sample language^a^
Preprints allowed	86	“Presentation of data at a scientific meeting, as a poster, abstract, orally, on a CD, or as an abstract on the web, or on a preprint server does not conflict with submission to the Lancet journals.”—*The Lancet *(https://www.thelancet.com) “Please note that preprints can be shared anywhere at any time, in line with Elsevier's sharing policy. Sharing your preprints e.g. on a preprint server will not count as prior publication.”—*Journal of Hepatology *(https://www.journal-of-hepatology.eu) “Posting of manuscripts on institutional websites or on recognized community preprint servers, such as bioRxiv, is permitted under our publication policy. Authors must retain copyright to such postings and are encouraged to contact the journal editors to discuss their specific manuscript if they have questions. **Please note that the AACR does not support posting of revised manuscripts that respond to editorial input and peer review or the final published version to preprint servers.**”^b^—*Cancer Discovery *(https://aacrjournals.org)
Case-by-case determination	13	“Public dissemination of manuscripts prior to, simultaneous with, or following submission to this journal, such as posting the manuscript on preprint servers or other repositories, **will necessitate making a determination of whether publication of the submitted manuscript will add meaningful new information to the medical literature or will be redundant** with information already disseminated with the posting of the preprint.”^b^—*JAMA *(https://jamanetwork.com/journals/jama)
Preprints prohibited	1	“Manuscripts that have been posted on a preprint server will NOT be considered by the Journal.”—*Journal of Allergy and Clinical Immunology *(https://www.jacionline.org)

^a^ Quotes are taken from each journal’s instructions for authors.

^b^ Emphasis was added by the authors for clarity. The quoted language is not the full text of each policy, but the relevant portion only.

**Table 2.** Median Impact Factor by Preprint Policy Category

Category	No. of journals	Impact factor, median (IQR)
Preprints allowed	86	12.94 (10.64-18.87)
Case-by-case determination	13	15.92 (12.00-20.77)
Preprints prohibited	1	NA

Abbreviations: IQR, interquartile range; NA, not applicable.

## Discussion

This cross-sectional study of preprint policies among the 100 clinical journals with the highest impact factors suggests that clinical journals are broadly supportive of considering clinical research preprints for publication. After classifying each journal according to the content of its preprint policies, we found that 86% of these journals allow for submitting articles previously posted as preprints. Our findings show that prohibitive journal preprint policies are in the minority, suggesting that clinical research journals are willing to consider manuscripts previously published on preprint servers. As a result, clinical researchers may feel less concerned that posting a manuscript on a preprint server will disqualify it from publication by a journal. However, the 13 journals whose policies were classified as case-by-case represent a potential barrier to researchers’ trust that manuscripts posted as preprints will be considered by clinical journals.

This study was limited to a search of publication policies at a single point in time, although journals may change their policies on preprints as preprint posting becomes more common. The COVID-19 pandemic has demonstrated the advantages of preprints, especially for rapid dissemination of information during a global health crisis, as well as the dangers that they may pose in disseminating false information. Preprints aim to bring expediency and transparency to clinical research, and current journal policies suggest that the community is open to their use.
